# Physio4FMD: protocol for a multicentre randomised controlled trial of specialist physiotherapy for functional motor disorder

**DOI:** 10.1186/s12883-019-1461-9

**Published:** 2019-10-21

**Authors:** Glenn Nielsen, Jon Stone, Marta Buszewicz, Alan Carson, Laura H. Goldstein, Kate Holt, Rachael Hunter, Jonathan Marsden, Louise Marston, Hayley Noble, Markus Reuber, Mark J. Edwards, on behalf of the Physio4FMD Collaborative Group, David Breen, Christine Burness, Hannah Callaghan, Jan Coebergh, Patrick Cookson, Paul Cooper, Lee Drake, Paula Gardiner, Thomas Gilbertson, Laura Irvine, Emily Jay, Uzma Khan, James Magro, Elizabeth Mallam, Luke Massey, Cameron Moss, Rachel Newby, Federico Ricciardi, Gillian Sare, Rhiannon Sears, Sumeet Singhal, Biba Stanton, Anna Stone, Ann-Marie Strudwick, Gillian Szeto, Tiago Teodoro, Volker Teweleit, Michael Walsh, Kathleen White, Mahinda Yogarajah

**Affiliations:** 1 Motor Control and Movement Disorders Group, Institute of Molecular and Clinical Sciences, St Georges University of London, London, UK; 2 Centre for Clinical Brain Sciences, University of Edinburgh, Royal Infirmary of Edinburgh, Edinburgh, UK; 3 Research Department of Primary Care and Population Health, UCL, London, UK; 4 Priment Clinical Trials Unit, UCL, London, UK; 5 King’s College London, Department of Psychology, Institute of Psychiatry, Psychology and Neuroscience, King’s College London, De Crespigny Park, London, UK; 6 School of Health Professions, University of Plymouth, Plymouth, UK; 7 Academic Neurology Unit, University of Sheffield, Royal Hallamshire Hospital, Sheffield, UK

**Keywords:** Physiotherapy, Physical therapy, Functional, Functional motor disorder, Conversion disorder, Psychogenic, Randomised controlled trial, Clinical trial

## Abstract

**Background:**

Patients with functional motor disorder (FMD) experience persistent and disabling neurological symptoms such as weakness, tremor, dystonia and disordered gait. Physiotherapy is usually considered an important part of treatment; however, sufficiently-powered controlled studies are lacking. Here we present the protocol of a randomised controlled trial (RCT) that aims to evaluate the clinical and cost effectiveness of a specialist physiotherapy programme for FMD.

**Methods/design:**

The trial is a pragmatic, multicentre, single blind parallel arm randomised controlled trial (RCT). 264 Adults with a clinically definite diagnosis of FMD will be recruited from neurology clinics and randomised to receive either the trial intervention (a specialist physiotherapy protocol) or treatment as usual control (referral to a community physiotherapy service suitable for people with neurological symptoms). Participants will be followed up at 6 and 12 months. The primary outcome is the Physical Function domain of the Short Form 36 questionnaire at 12 months. Secondary domains of measurement will include participant perception of change, mobility, health-related quality of life, health service utilisation, anxiety and depression. Health economic analysis will evaluate the cost impact of trial and control interventions from a health and social care perspective as well as societal perspective.

**Discussion:**

This trial will be the first adequately-powered RCT of physical-based rehabilitation for FMD.

**Trial registration:**

International Standard Randomised Controlled Trials Number ISRCTN56136713. Registered 27 March 2018.

## Background

Functional motor disorder (FMD) can be defined as neurological symptoms affecting movement that are caused by loss of control or agency over movement, rather than a structural disease process. FMD is the motor-dominant variant of functional neurological disorder (also known as conversion disorder) [1]. Typical presentations of FMD include weakness, tremor, jerks, dystonia, gait disorder, or a combination of these symptoms. Most patients also experience non-motor functional neurological symptoms, such as sensory disturbance, memory complaints, pain, fatigue or dissociative seizures [1]. It is also common for patients with FMD to have comorbid health problems such as neurological disease [2, 3]. FMD is often described as a condition at the interface between neurology and psychiatry.

The incidence of FMD is reported to be in the range of 4 to 12 per 100,000 [4], making it similar in incidence to multiple sclerosis and Parkinson’s disease. The long-term outcome is variable but often poor. A systematic review of long term follow up studies found that approximately 40% of patients were the same or worse at an average of 7 years and the majority of patients remained symptomatic [5]. However, little is known about the outcome of patients who receive timely specialist interventions.

In recent years, specialist physiotherapy has emerged as a promising treatment for FMD [6]. A number of cohort studies report positive results from physical interventions that are based on a biopsychosocial understanding of FMD and encompass psychosocial therapeutic elements [7–10]. The first controlled trial of physical-based rehabilitation for FMD, published in 2014, compared an intervention group with patients waiting for treatment [11]. In this study 60 patients with a functional gait disorder were randomised to a 3-week inpatient physical rehabilitation programme or a 4-week waiting list. Group comparisons demonstrated a statistically significant improvement with treatment across a range of physical and quality of life outcome measures. The mean differences immediately after the intervention were 6.9 units in the Functional Mobility Scale (15 point range), 8.4 Functional Independence Scale units (108 point range), and 11.7 SF12 Physical Domain units (maximum score 100). Post intervention improvement was sustained at 12 months follow up, except for the SF12 Mental Health domain which showed an immediate treatment effect of 6.7 units (100 point range) but was no longer statistically different from baseline scores at 12 months.

Our group has recently completed a single centre, randomised feasibility trial of specialist physiotherapy for FMD [12]. The intervention was based on a consensus recommendation paper describing physiotherapy for FMD [13] and had been previously tested in a small prospective cohort study (*n* = 47) [9]. This intervention was developed using a novel “movement retraining” model, harnessing and making explicit the internal inconsistencies seen in FMD which are used to make the diagnosis. For instance, an inability to carry out volitional movements (e.g. active ankle dorsiflexion) with retained ability to carry out movements of the same muscle groups in a different context (e.g. retained ability to activate dorsiflexor muscles by standing on their heels). The feasibility trial randomised 60 patients to either our specific specialised physiotherapy protocol for FMD or a treatment as usual control (consisting of referral to standard community neuro-physiotherapy). Participants were followed up at 6 months. The intervention was considered unsuitable for people whose primary problem (above motor symptoms) was pain, fatigue or dissociative seizures, and such patients were excluded from the study. We also excluded people who had psychiatric comorbidity (such as anxiety or depression) that was deemed to require assessment and treatment before starting physiotherapy. 32% of patients with FMD seen in the recruiting neurology clinics met the inclusion criteria. 90% of this group consented to participate in the trial and only 5% were lost to follow up. Participants rated the intervention as highly acceptable.

As part of the feasibility trial we tested a range of physical, mental health and quality of life outcome measures. At 6 month follow up, the intervention group scored higher on measures of physical function but there were no differences in scores of mental health. The Short Form 36 (SF36) Physical Function domain showed a mean difference between groups of 19.8 (95% CI 10.2, 29.5, Cohen’s d = 0.7), after adjusting for baseline scores. In a patient-rated 5-point Likert scale of impression of change, 72% of the intervention group rated their symptoms as improved at 6 months, compared to 18% in the control group. These promising results highlight the importance of progressing this research and conducting an adequately powered randomised trial.

In summary, there are a number of small studies showing promise that specialist physiotherapy is effective for people with FMD, however, there is a lack of evidence from adequately-powered randomised controlled trials (RCTs). Here we report the protocol for a RCT of specialist physiotherapy for functional motor disorder (Physio4FMD). In work leading up to this research, we developed consensus recommendations for physiotherapy treatment [13]. The trial intervention was based on these recommendations and further developed during the course of our clinical practice and clinical research [9, 12]. In line with MRC guidelines for evaluating complex interventions [14], we have completed proof of principle in a feasibility trial, obtaining preliminary evidence for efficacy in a small sample. The next stage is to evaluate the clinical and cost effectiveness, as well as generalisability of the intervention in a pragmatic multicentre RCT.

## Aims and objectives

The overall aim of the Physio4FMD trial is to evaluate the clinical and cost effectiveness of a specialist physiotherapy protocol for FMD, compared to treatment as usual within a pragmatic, multicentre RCT. The primary objective is to evaluate the effectiveness of specialist physiotherapy compared to treatment as usual in reducing disability, measured by the Physical Function domain of the SF36 at 12 months post randomisation.

The secondary objectives are to evaluate the effectiveness of the specialist physiotherapy protocol compared to treatment as usual on the following domains of measurement:

1. The patient’s perception of change in their functional motor disorder
2. Level of mobility
3. Health-related quality of life
4. Employment and return to work
5. Objective measures of health service use
6. Subjective measures of health service use
7. Understanding and illness beliefs
8. Anxiety and depression
9. Satisfaction with treatment
10. Confidence that the diagnosis of FMD is correct
11. The influence of the number of somatic symptoms reported at baseline on treatment outcome

In addition, the cost-effectiveness of specialist physiotherapy compared to treatment as usual will be evaluated in a comprehensive health economic analysis.

## Methods and design

### Trial design

**Fig. 1:**
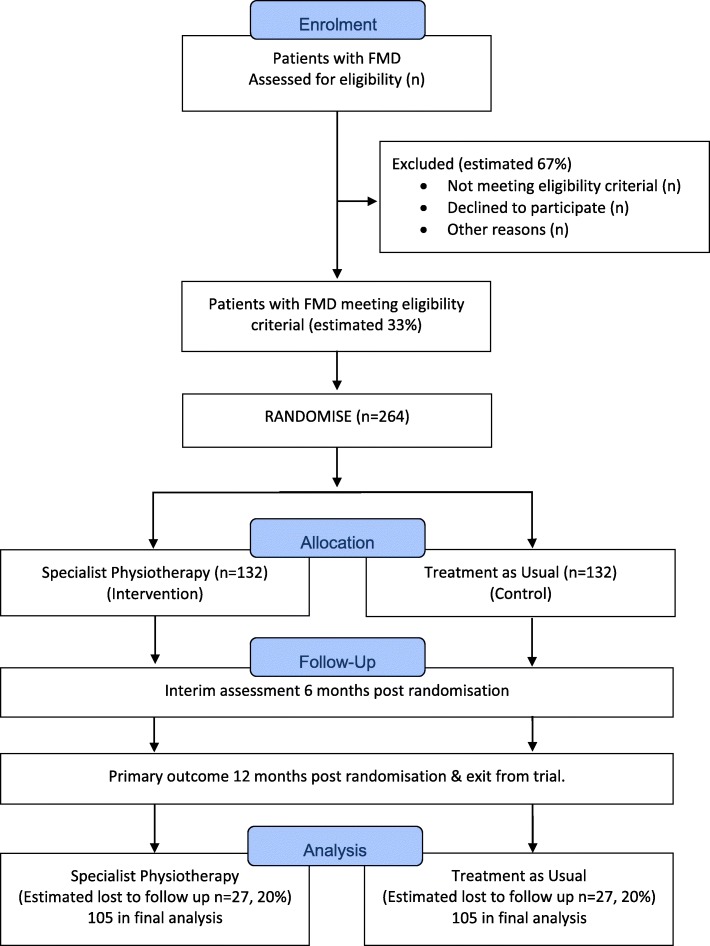
CONSORT flow diagram

The study design is a pragmatic, UK multicentre, single-blind, parallel group RCT in adults with FMD. The trial will compare a specialist physiotherapy protocol with treatment as usual, which is defined in the trial as a referral to a community physiotherapy service suitable for people with neurological symptoms. Participants are randomised with a 1:1 ratio to either treatment arm and will be assessed at baseline (pre-randomisation), 6 and 12 months post-randomisation. The primary outcome is assessed at 12 months. The trial flowchart is presented in Fig. 1.

### Participants

The target population is adults with a “clinically definite” diagnosis of functional motor disorder [15].

### Recruitment

The neurologists signed up to the trial will screen outpatients and inpatients (due to be discharged) who have been referred to them. It is the responsibility of the neurologist to determine a patient’s eligibility for the trial. As per usual practice, the diagnosis of FMD will be explained to the patient following a standardised method which emphasises the diagnosis based on positive clinical features, potential for reversibility and that psychological comorbidity, if present, is not part of the diagnosis [16]. Patients meeting the eligibility criteria will be informed about the study by the neurologist and provided with a patient information sheet. The neurologist will then seek agreement from the patient to be contacted by a member of the research team. Those willing to take part in the trial will be invited to an appointment to provide informed consent and complete baseline assessments before being randomised to their treatment allocation.

### Inclusion criteria

The inclusion criteria are:

1. New or returning patients presenting to participating outpatient neurology clinics and neurology inpatients.
2. The patient has a “clinically definite” diagnosis of FMD according to the Gupta and Lang diagnostic classification criteria [15].
3. Age 18 or over.
4. Diagnostic investigations have come to an end.
5. The patient is accepting of the intervention.
6. Motor symptoms must be sufficient to cause significant distress or impairment in social, occupational or other important areas of functioning (subjectively described by the patient), independent of other comorbidities.

### Exclusion criteria

Participants will be excluded from the trial if:

1. The recruiting neurologist deems the patient to have severe psychiatric comorbidity, including factitious disorder, self-harm, anxiety and depression, which would interfere with the patient’s ability to participate in physiotherapy.*
2. The patient has an organic diagnosis which explains the majority of their symptoms or disability.
3. The patient has pain, fatigue or dissociative seizures that would interfere with their ability to engage in the trial physiotherapy intervention.**
4. Disability to the extent that the patient requires assistance for toileting.
5. The patient is unable to attend 9 sessions of physiotherapy over a 3-week period, within 6 weeks of their initial neurology consultation.
6. Ongoing unresolved compensation claim or litigation.
7. The patient has no fixed address or is seeking rehousing through their council for disability access reasons.
8. Unable to understand English sufficiently to complete questionnaires.
9. The patient has a documented learning disability that prevents them from answering questionnaires independently.
10. The patient lacks capacity to give consent.

* The decision to exclude a patient due to psychiatric comorbidity is a clinical decision made by the neurologist, rather than a decision based on a screening tool or questionnaire. We believe that no single screening tool or questionnaire would serve this purpose. Additionally, there is insufficient data on which to base cut-off scores to exclude patients on any particular questionnaire.

** Pain, fatigue and dissociative seizures are not absolute exclusions. Based on previous research we expect most participants to experience persistent pain and fatigue to various degrees. The participant is only excluded if these symptoms are judged to be likely to prevent them from engaging with the intervention.

### Interventions

#### Specialist physiotherapy (intervention group)

Participants in this group will receive a protocolised, specialist physiotherapy programme that has been designed based on a specific and novel biopsychosocial understanding of FMD [17, 18]. This understanding emphasises the role of self-focused attention in driving symptoms and this is addressed as part of the intervention by (i) helping the patient to understand the role of attention and; (ii) movement retraining with an external focus of attention (implicit motor learning). These are important ways in which the intervention differs from typical neurorehabilitation, which usually involves explicit motor learning strategies and exercises that encourage attention to be directed towards movement and sensations. See Table 1 for a description of the intervention following the TIDIER framework [19]. The intervention is also based on published consensus recommendations for physiotherapy treatment of FMD [13]. The key elements of the intervention are (i) symptom education; (ii) movement retraining with redirection of the patient’s focus of attention; and (iii) developing a long term symptom management plan. The intervention is delivered over 9 sessions within a 3-week period, plus a 3-month follow up session. Two sessions may be scheduled in 1 day if separated by a break. Flexibility is allowed in the arrangement of sessions over the 3 weeks to accommodate other commitments of the patient and physiotherapist.

**Table 1:** Physio4FMD Intervention description following the TIDIER checklist

1. Name	Provide the name or a phrase that describes the intervention.
	Physio4FMD: Specialist physiotherapy for functional neurological disorder.
2. Why	Describe the rationale, theory, or goal of the elements essential to the intervention.
	The rationale for the Physio4FMD treatment is primarily based on a particular aetiological model for FMD [18]. The model highlights two key mechanisms that drive functional motor symptoms. These are: 1. Functional motor symptoms require the patient’s attention, at a level without voluntary control, to be directed towards their body in order to manifest. When the patient’s attention is distracted, the movement disorder disappears or dampens. 2. The patient has an expectation, at a level without voluntary control, that their movement will be abnormal; this expectation is associated with a particular illness belief (e.g. my legs are paralysed). Expectations of abnormal movement influence motor output with symptoms arising as a ‘habit’ that the nervous system has got in to. The Physio4FMD intervention addresses attention-related movement problems by retraining activity (movement) while redirecting the patient’s focus of motor attention. Altered expectations and illness beliefs are addressed through education, demonstrating to the patient that they can move normally and helping the patient to develop strategies that normalises their movement during every day activities. The essential elements of the intervention are: 1. Prior to physiotherapy, the participant receives a diagnosis of FMD by a neurologist. The neurologist gives a thorough explanation of FMD and how the diagnosis was made positively based on clinical features, and not as a diagnosis of exclusion. 2. Education about FMD, following which the participant and physiotherapist collaboratively devise a formulation to theorise how the patient developed their movement problem using the aetiological model as a framework [9]. 3. Education about common problems associated with FMD (persistent pain, fatigue and memory/concentration problems). 4. Movement and posture retraining, with the participant’s focus of attention directed away from their body (areas addressed include sitting postures, sit to stand, walking, getting on and off the floor, stairs, upper limb problems, and use of walking aids). 5. Developing a self-management plan (which includes understanding medication, addressing boom-bust patterns of activity, how to incorporate movement strategies into daily routine, self-management goals, and managing symptom exacerbations and relapses).
3. What: Materials	Describe any physical or informational materials used in the intervention, including those provided to participants or used in intervention delivery or in training of intervention providers. Provide information on where the materials can be accessed (such as online appendix, URL).
	Information for Neurologists, document: Each trial neurologist will receive a document summarising their role in the trial, which includes an explanation of how to apply the eligibility criteria, how to explain FMD to patients, how to discuss the trial with potential participants, and requirements for follow up. Patient Workbook: Each intervention participant is given a workbook, which guides the intervention. The workbook is completed by both the participant and the physiotherapist during treatment. Key sections of the workbook are: (i) understanding the diagnosis; (ii) neuroanatomy and physiology; (iii) pages for participants to reflection on sessions; (iv) analysis and exploration of movement; (v) movement retraining; (vi) understanding problems associated with FMD (pain, fatigue and memory problems); and (vii) self-management plan. Amongst other goals, the workbook helps to standardise the intervention. Physiotherapy Intervention Manual: Each physiotherapist providing the trial intervention will receive an intervention manual that complements the Physio4FMD training programme.
4. What: Procedures	Describe each of the procedures, activities and/or processes used in the intervention, including any enabling or support activities.
	Neurology: Prior to enrolling in the trial, participants in both groups are seen by one of the study neurologists. The diagnosis of FMD is made and explained to the patient following a standardized explanation [16]. Participants in both arms of the trial will be followed up by their neurologist at least once within 12 months of their initial neurology consultation. Physiotherapy – Education: Participants receive a standardised explanation of FMD using the workbook as a guide. This is followed by an individualised formulation, where the participant and physiotherapist collaboratively devise a theoretical explanation for how the person came to develop FMD, using a symptom model [9]. The formulation seeks to determine relevant risk factors, triggers, initial symptoms, examples of attention affecting movement, adaptive coping strategies, secondary changes, and social factors. Education includes information about some common problems associated with FMD (pain, fatigue, and memory/concentration). Physiotherapy – Movement Retraining**:** Movement retraining generally follows a sequential motor learning approach, building up desired movement patterns starting from elementary, symptom free components of movement [13]. Problematic movement patterns and tasks are identified in the initial assessment; only those relevant to individual are retrained. The workbook prompts exploration and practice of 7 key tasks (i) sitting postures, (ii) sit to stand, (iii) standing and walking, (iv) arm and hand problems, (v) use of walking aids, (vi) getting on and off the floor, and (vii) using stairs. Movement retraining is tailored to the individual, but should adhere to the key principle of employing strategies that redirect the patient’s focus of motor attention. In practice this is achieved by: • Asking the patient to focus on the goal of the task rather than the mechanics of movement • Practice movements in front of a mirror (the patient focus of attention is redirected externally to their reflection) • Redirecting the patient’s focus to an another part of their body or a specific component of the movement Specific exercises and activities to retrain movement that conform to the above principles are suggested in the intervention manual and have been published elsewhere [13]. If available, the physiotherapist may choose to use the following standard physiotherapy adjuncts: electrical muscle stimulation, treadmill, other exercise equipment. Physiotherapy – Personal Reflections: At the end of each physiotherapy session, the participant is encouraged to write a reflection in their workbook, addressing several prompts. The subsequent session starts by reviewing the reflection of the previous session and discussing any questions or issues that arise. After which, a plan is made for the current session. Physiotherapy – Self-Management: To conclude treatment, a personalised self-management plan is developed, which usually includes: (i) a summary of useful strategies that help to normalise movement; (ii) activity plans to address boom and bust patterns and how to progress activity; (iii) future goals; and (iv) what to do on difficult days and during periods of symptom exacerbation.
5. Who provided	For each category of intervention provider, describe their expertise, background and any specific training given.
	Neurologists: All neurologists involved in the trial will be employed at a consultant level at one of the trial sites. Only neurologists with a clinical interest and experience in treating patients with FMD will be invited to participate. They will receive training from one of the research neurologists (ME or JS) in person or by telephone, lasting 30–60 min. The training topics are listed in item 3 above. This information will be supplemented with written information. Physiotherapists: The intervention physiotherapists will have at least 2 years’ experience working in the field of neurological physiotherapy. Each will undergo 1 week full time training, delivered by the research physiotherapists (GN and KH). Competency will be assessed according to a checklist that ensures the physiotherapist has demonstrated an understanding or proficiency in delivering the key ingredients of the intervention. They will also receive a comprehensive intervention manual. During delivery of the intervention, each physiotherapist will receive supervision over telephone from one of the research physiotherapists. At least one supervision session will be planned for every intervention participant treated.
6. How	Describe the modes of delivery (such as face to face or by some other mechanism, such as internet or telephone) of the intervention and whether it was provided individual or in a group.
	Each session is conducted face to face and individually (there are no group treatment sessions).
7. Where	Describe the type(s) of location(s) where the intervention occurred, including any necessary infrastructure or relevant features.
	Participants will be recruited from inpatient or outpatient neurology clinics. The physiotherapy sessions will be held in a physiotherapy gym or clinic with space suitable for movement and gait retraining and space suitable for education and writing in the intervention workbook. The only essential equipment is a full-length mirror. Physiotherapists can make use of other standard therapeutic equipment as appropriate (e.g. treadmill, electrical muscle stimulation device, other exercise equipment).
8. When and how much	Describe the number of times the intervention was delivered an over what period of time including the number of sessions, their schedule and their duration, intensity or dose.
	The physiotherapy intervention is delivered over 9 sessions, which should be completed within a 3-week period. There is also a 3-month follow up session. Each session should last between 45 min and one hour. It is permissible to schedule 2 sessions in 1day, separated by a (lunch) break. Home exercise programmes are not usually part of the intervention. Instead, the patient is encouraged to incorporate movement strategies and plans (e.g. activity plan to avoid boom and bust patterns) into their normal daily routine.
9. Tailoring	If the intervention was planned to be personalised, titrated or adapted, then describe what, why, when and how.
	The intervention is standardised by following a workbook; however, only information and tasks relevant to the individual’s problem will be addressed. Movement retraining focuses on 7 key tasks, which are described in item 4 above. When retraining each task, strategies are adapted and personalised for the individual, but the approach should adhere to the key principle of redirecting the participant’s attention away from their movement or body. Passive interventions such as massage and acupuncture are discouraged.
10. Modifications	If the intervention was modified during the course of the study, describe the changes (what, why, when, and how).
	Not applicable.
11. How well: Planned	If intervention adherence or fidelity was assessed, describe how and by whom, and if any strategies were used to maintain or improve fidelity, describe them.
	Fidelity of the intervention will be assessed in the following ways. (i) At the level of the physiotherapist: The physiotherapist providing the trial intervention will complete a treatment checklist (paper form) for each participant, which conforms to the TIDIER intervention description. (ii) At the level of the participant: We will monitor the content, length and number of physiotherapy sessions by participant report for both trial arms with a structured telephone survey. The interview will also assess for contamination between the groups. (iii) Fidelity of the trial intervention will also be assessed by evaluating a random sample of completed intervention workbooks. The workbook guides the intervention and is filled in during the treatment session by both the participant and physiotherapist. It therefore provides a record of the content of sessions. Fidelity will be judged against predefined criteria. We aim to assess 40% of the intervention workbooks.
12. How well: Actual	If intervention adherence or fidelity was assessed, describe the extent to which the intervention was delivered as planned.
	Not applicable.

The intervention is guided by a workbook that is completed by both the patient and physiotherapist during sessions and the patient is encouraged to write a reflection at the end of each day. The intervention starts by taking a full history from the patient and completing a physical assessment. This is followed by education about FMD according to a specific biopsychosocial aetiological model [9]. The patient and physiotherapist then collaboratively devise a formulation to theorise how the patient developed their movement problem using the biopsychosocial model as a framework [9]. It takes into account risk factors, triggering events, psychological factors (such as panic at onset), self-focused attention disrupting normal movement, and secondary problems (such as unhelpful reinforcement of symptomatic movement patterns). Movement retraining mostly occurs within the context of tasks such as standing up, sitting down, walking, drinking from a cup, etc. It generally follows a sequential motor learning approach, where elementary symptom-free components of movement are established and then built upon in successive stages to reshape normal movement patterns. Strategies that normalise movement (for example, redirecting the patient’s focus of motor attention away from the body) are incorporated into movement retraining. Examples of such strategies have been described in detail elsewhere [13]. Movement retraining is practised and progressed over the remaining sessions, interspersed with information about managing pain, fatigue and concentration problems, if they are relevant to the individual. A long-term self-management plan is completed in the workbook in the final sessions. The 3 month follow up session is an opportunity to review and update the self-management plan, as well as to provide encouragement and reassurance.

The physiotherapists delivering the trial-intervention will have at least 2 years’ experience working in neurorehabilitation. They will receive a manual describing the intervention and will undergo a comprehensive training programme delivered over 5 consecutive days. They will also receive a supervisory phone call from an experienced member of the trial team for each participant treated in the trial, which aims to provide clinical support, as well as ensuring fidelity with the trial intervention.

#### Treatment as usual physiotherapy (control group)

The control arm of the trial is “treatment as usual”, which, for the purposes of this trial, is a referral to a community physiotherapy service appropriate for patients with neurological symptoms. The referral letter to physiotherapy will come from the diagnosing neurologist. It will state the diagnosis and that the patient may benefit from physiotherapy treatment. The referral letter will be accompanied by a copy of the patient’s neurology consultation letter.

We will monitor the content of the control physiotherapy arm via participant report in a telephone survey. There are no formal evidence-based guidelines for physiotherapy for FMD, therefore the treatment received by the control participants will be variable. Based on the preceding feasibility trial we expect that most physiotherapists will provide a combination of gait retraining, stair practice, balance, non-specific cardiovascular exercise, specific strengthening exercises, stretching, and provision of walking aids or splints. The frequency and number of physiotherapy sessions provided by community therapy services will differ between centres, according to local policies. In addition, some trial participants may also be offered treatment from occupational therapy and/or clinical psychology, although in our feasibility trial we found that this was rare. Additional treatments such as these will be recorded at the 6 month and 12 month data collection (via the patient self-complete Client Service Receipt Inventory).

We anticipate that, in general, the intervention will differ from the control treatment in the following ways: (i) greater emphasis on education using a specific aetiological model; (ii) movement retraining will focus on redirecting attention away from the movement; (iii) greater emphasis on self-management; (iv) greater number of sessions; (v) higher intensity or frequency of sessions; (vi) use of FMD specific written materials (i.e. workbook); (vii) acknowledging and addressing coexisting problems (i.e. pain, fatigue, and concentration). We will examine these assumptions in a post-treatment telephone survey of participants from both groups.

#### Both groups

Participants in both arms of the trial will be followed up by their neurologist at least once within 12 months of their initial neurology consultation as part of standard NHS care.

To encourage retention, we will reimburse the cost of travel to research appointments and physiotherapy treatment, up to the value of £25 per appointment.

### Outcome measures

Outcome measures will be collected at 6 and 12 months’ post randomisation. The primary outcome is the Physical Function domain of the SF36 questionnaire [20, 21], measured at 12 months post randomisation. The secondary outcome measures and timing of data collection are listed in Table 2.

**Table 2:** Outcome Measures

Assessment	Domain of measurement	Timing		
		T0	T1	T2
Physical Function domain, SF36 [21]	Physical disability	X	X	X
Short Form 36 (SF36) [20]	Health related quality of life	X	X	X
Functional Mobility Scale [22]	Mobility related disability	X	X	X
Revised Illness Perception Questionnaire [23]	Illness belief and understanding	X	X	X
Hospital Anxiety and Depression Scale [24]	Anxiety and Depression	X	X	X
Clinical Global Impression Scale of Improvement (CGI-I) [25]	Patient perception of change		X	X
Fatigue (5-point scale) [26]	Fatigue	X	X	X
EQ-5D-5 L [27]	Health Economics, to generate QALYS	X	X	X
Client Service Receipt Inventory (CSRI) [28]	Health Economics, health resource use	X	X	X
Work Productivity & Activity Impairment Questionnaire (WPAI) [29]	Employment and return to work	X	X	X
Confidence in correctness of diagnosis of FMD (10 point scale) [30]	Illness belief, confidence in diagnosis	X	X	X
Extended Patient Health Questionnaire-15 (PHQ-15) [31, 32]	Somatic symptom severity	X		
Hospital Episode Statistics (HES)	Health Economics, health resource use	X		X

*Abbreviations*: *T0* Baseline assessment, *T1* 6-month assessment, *T2* 12-month assessment, *QALYS* Quality Adjusted Life Years. Pain is assessed as part of the SF36 and EQ-5D-5L questionnaires

Hospital Episode Statistics (HES) covering acute hospital care (inpatient and outpatient attendances) from NHS Digital and the equivalent data from NHS Scotland (eDRIS) will be used as an objective (non-patient reported) measure of change, comparing the difference between groups at 12 months, adjusting for baseline data. The Extended Patient Health Questionnaire-15 (PHQ-15) [31, 32] will be collected at baseline only. This data will be used to explore the relationship between somatic symptoms reported at baseline with outcome.

### Other demographic and clinical data

At baseline we will collect a range of data to describe the study population. Demographic information will include: age, gender, ethnicity, marital status, living arrangements, dependents, education, and employment status. Clinical information will include: symptom phenotype, symptom duration, past medical history, and previous treatments. Information about adverse events will be collected systematically during a post-treatment telephone assessment and the self-complete 6 and 12 month follow up assessments. Participants are also instructed to inform the trial team about adverse events as soon as possible as they occur. The schedule of data collection is displayed in Table 3.

**Table 3:** Schedule of data collection

Study Procedures		Face-to-face assessment	Post Treatment telephone call	Telephone, mail or online form assessment*	
		Screening & Baseline Assessment		6 Months	12 Months
Informed consent		✓			
CRF	Inclusion/exclusion criteria	✓			
	Medical history	✓			
	Demographics	✓			
	Clinical characteristics	✓			
Assessments	Short Form 36	✓		✓	✓
	Functional Mobility Scale	✓		✓	✓
	Revised Illness Perception Questionnaire	✓		✓	✓
	Hospital Anxiety & Depression Scale	✓		✓	✓
	Client Service Receipt Inventory	✓		✓	✓
	EQ-5D-5 L	✓		✓	✓
	Work Productivity & Impairment Questionnaire	✓		✓	✓
	Clinical Global Impression Scale (CGI-I)		✓	✓	✓
	Fatigue State	✓	✓	✓	✓
	Confidence in correctness of diagnosis	✓		✓	✓
	Extended Patient Health Questionnaire-15	✓			
Randomisation		✓			
Adverse events screen			✓	✓	✓
Satisfaction with intervention Questionnaire			✓		
Participant description of intervention			✓		
HES data obtained from NHS Digital and eDRIS services covering the previous 18 months					✓

*6 and 12 month follow up assessments will be completed by the participant's preferred option out of telephone, mail or online form

The fidelity of the intervention will be assessed in the following ways:

(i) Both groups: We will monitor the provision of physiotherapy in both groups by participant report with a structured telephone survey. Participants will be surveyed by a nonblinded member of the research team as soon as possible after completing treatment. The survey will explore the content, number and length of physiotherapy sessions, as well as the participant’s satisfaction with their allocated treatment.
(ii) Intervention group: The physiotherapist providing the study intervention will complete a treatment checklist (paper form) for every participant, recording the content of each session. The checklist is based on the TIDIER checklist description of the intervention, (see Table 1) [19].
(iii) Intervention group: We will assess a random sample of intervention workbooks. The workbook guides the intervention and is completed during the treatment session by both the participant and physiotherapist. It therefore provides a record of the content of sessions. Two workbooks will be randomly selected from every 5 participants treated by each intervention physiotherapist (40% of intervention participants). The workbooks will be assessed against a predetermined set of criteria to determine the extent to which treatment followed the intervention protocol.

### Sample size calculation

The power calculation was based on data from the preceding single centre randomised feasibility trial [12], in which, at 6 months follow up we found a between groups difference of 19.8 in the SF36 Physical Function domain in favour of the intervention group (after adjusting for baseline differences). A treatment effect is likely to be smaller in a pragmatic multicentre trial; we therefore aim to detect a 9 point difference in the SF36 Physical Function domain, which we consider to be a clinically important difference.

In order to detect a 9 point difference in the SF36 Physical Function domain at the 5% level of significance with 90% power, we require 264 participants, 132/group. The calculation used the ANCOVA method with one pre and one post randomisation measurement, assuming a standard deviation of 22, which gave an estimated sample size of 75/group. The sample size was inflated to account for therapist effect (clustering) in the intervention arm, assuming eight therapists. We assumed the average number of participants treated by physiotherapists delivering the trial intervention (cluster size) will be 9 after 20% drop out. The resulting inflation factor was 1.4, which we applied to both groups to give equal group sizes of 105/group. The sample size was then inflated by a factor of 1.25 to account for a 20% dropout rate.

### Randomisation and blinding

Randomisation will be conducted by the Trial Manager using a remote web-based application, Sealed Envelope [33], and will be overseen by a clinical trials unit (UCL Priment). Randomisation will occur at the level of the participant, stratified by site. Block randomisation with random block sizes will be used to ensure even allocation of intervention and control participants across sites.

The researchers collecting outcome data, the statisticians and health economists will be blind to treatment allocation. The Trial Manager, participants and treating clinicians will not be blinded due to practical reasons.

### Statistical analysis

An intention to treat analysis will be conducted after the database is locked following collection of final 12 month follow up data. The primary outcome, the SF36 Physical Function domain, will be analysed using random effects modelling, with therapist as the random effect (individuals for those in the control group), controlling for baseline scores. The Clinical Global Impression Scale will be collapsed into two groups, good outcome and poor outcome. Good outcome will be defined as ratings of “much improved” or “improved” and poor outcome will be defined as rating of “same”, “worse”, or “much worse”. This will be analysed using random effects logistic regression. Other clinical secondary outcomes will be analysed as for the primary outcome. We will perform sensitivity analyses looking at the effect of missing data, additional interventions received (e.g. psychology) and dose-response relationship for the control and intervention conditions.

We aim to complete an exploratory analysis of prognostic indicators. This will use random effects logistic regression modeling to determine predictors of a good or bad outcome from baseline demographic and clinical characteristics. Outcome will be determined by a self-rating of “improved” or “much improved” on the CGI-I scale and a 10 point increase in SF36 Physical Function domain score. This analysis will be indicative, and any factors which appear to be associated with the outcome will need further investigation in a study that is powered for that purpose.

### Health economic analysis

The aims of the health economic evaluation will be twofold: (i) To estimate the cost impact of the Specialist Physiotherapy protocol compared to treatment as usual for FMD over 12 months, firstly from a health and social care cost perspective and secondly from a societal perspective. (ii) Calculation of Quality Adjusted Life Years (QALYs) over 12 months from responses to the EQ-5D-5 L and calculated as the area under the curve adjusting for baseline [34]. This will be used to calculate the mean incremental cost per QALY gained with the specialist physiotherapy protocol compared to treatment as usual over 12 months. Bootstrapping will be used to construct confidence intervals, cost-effectiveness planes and cost-effectiveness acceptability curves. Sensitivity analyses will be conducted to test the impact of any assumptions made as part of the analysis. The primary health economic analysis will be from a health and social care cost perspective with a secondary analysis to account for the impact on employment from a societal perspective. Similar to the analysis of the primary outcome, we will use random effects modelling for the therapist effect.

An adapted version of the Client Service Receipt Inventory (CSRI), informed by our experience of using this questionnaire in the feasibility trial, will be used to collect resource use and employment information. The Work Productivity & Activity Impairment Questionnaire will be used to calculate the cost impact of improved engagement with employment as a result of being randomised to specialist physiotherapy. Productivity will be costed using the human capital approach. Other resource use will be costed using nationally published sources including the Personal and Social Services Research Unit [34], British National Formulary [35] and National Reference Costs [36].

For the HES data, we will report descriptive statistics for each service type (outpatient, A&E, inpatient) separately. Suitable descriptive statistics and statistical tests will be selected for each service type depending on the distribution of the data (i.e. non parametric tests for highly skewed data). We will include an analysis using general linear models (GLM) and appropriate family and log links to account for the distribution of the data. The GLM models will be used to calculate differences in service use between trial arms, adjusting for baseline service use. HES data will also be used to validate the results of the analysis of secondary/hospital care service use reported in the CSRI. The data will include information on Healthcare Resource Groups for inpatient HES data and diagnostic and procedural codes for all other data and will be costed using the National Reference Costs [36]. The CSRI analysis will be validated with the HES data by (i) applying more specific costs based on reason of attendance; (ii) checking the reliability of patient reporting; and (iii) investigating the implications for the cost-effectiveness analysis by including HES data for participants with missing data or for those lost to follow up.

### Data handling and monitoring

The trial will be overseen by and run according to the Priment Clinical Trials Unit standard operating procedures, ensuring the trial complies with Good Clinical Practice and maintains scientific integrity. The Trial Manager will monitor the accuracy of data entry throughout the trial and undertake source verification checking against paper records.

Baseline data and questionnaires will be collected on paper case report forms and entered into a web-based data management system, Sealed Envelope [33]. The 6 and 12 month assessments will be completed by participants according to their preferred method, either an online form, paper form, or with support from a research assistant over telephone. A non-contingent gift voucher will be sent to participants prior to 6 and 12 month data collection to encourage participation and minimise missing data (£10 at 6-months, £15 at 12-months).

Trial oversight committees will be set up according the requirements of the funder. A Trial Management Group, consisting of the grant holders, trial statistician and health economist and two patient and public representatives will meet regularly to monitor the conduct of the trial. An independent Trial Steering Committee (TSC) will meet six monthly to oversee the conduct of the trial. The TSC will be chaired by an independent research expert and consist of a statistician, health economist, an expert clinician and 2 patient and public representatives. An independent Data Monitoring and Ethics Committee (DMEC), consisting of two expert clinicians and a statistician will meet 6 monthly to review trial data and safety related issues/adverse events. The DMEC will advise the TSC.

### Ethical approval

Ethics approval was obtained from the London-Surrey Borders Research Ethics Committee, reference number 18/LO/0486. Approval was granted 28 March 2018. Local NHS approvals are being obtained prior to opening each trial site.

## Discussion

To our knowledge, this will be the first adequately powered, parallel group design RCT of physiotherapy or physical rehabilitation for FMD. The trial compares a protocolised specialist physiotherapy intervention with treatment as usual. The primary outcome is physical disability at 12 months post randomisation, as measured by the SF36 Physical Function domain. We will also assess a range of secondary outcomes and complete a comprehensive health economic analysis.

We support the choice of our primary outcome because the SF36 Physical Function domain has been found to be a valid measure of physical and mobility related disability [21]. While it is a self-report measure, it asks respondents to report limitations on their ability to carry out particular activities rather than the level of distress associated with these. The physical functions sampled include vigorous activities (such as participating in strenuous sports); moderate activities (such as pushing a vacuum cleaner); carrying groceries; climbing stairs; walking 100 yards/several hundred yards/more than a mile; and bathing or dressing. Poor Physical Function domain scores have been found to be related to low scores of physical performance tests such as low grip strength and longer Timed Up and Go test scores [21].

Our trial has a pragmatic design, in that it is conducted and contained within the real-world limitations of NHS pathways. The results of pragmatically-designed trials are considered to have greater generalisability than research conducted under highly controlled ideal conditions [37]. The pragmatic nature of our trial has implications that are both strengths and limitations of the trial design. One limitation of our pragmatic design is the ‘treatment as usual’ control condition. In the NHS there are no formal evidence-based guidelines for the treatment of FMD; therefore, we expect large variations in the number of sessions and the content of physiotherapy treatment received by control participants. We will monitor the provision of control physiotherapy (by participant report), and in the post hoc analysis we will explore the impact of the number of sessions received by control participants on treatment outcome. We considered trying to standardise the control intervention; however, this was not possible within our budgetary constraints.

The pragmatic nature of our trial gives rise to a number of design strengths. We have a relatively relaxed eligibility criteria that reflects the heterogeneity of people with FMD. Although we expect that only 25–30% of patients with FMD are suitable for a physiotherapy-led intervention (and will therefore meet our eligibility criteria), we have no restrictions on phenotype, age or symptom duration. The intervention is delivered within current NHS services by clinicians who would normally be involved with this patient group. Thus our outcomes should be considered generalisable.

The outcome measures are largely patient-reported subjective outcomes (with the exception of the HES data (Hospital Episode Statistics, linked to individuals), which will be reported for each participant and will be used to compare episodes of NHS care pre- and post-treatment). Objective measurement of symptoms and disability in FMD is a complex issue. Snapshot tests (such as dynamometry, gait speed, and movement disorder severity) have questionable reliability and validity as physical examination, is by definition, internally inconsistent in patients with FMDs [38, 39]. This is related to the mechanistic role of self-directed attention in driving the motor symptoms [18]. Arguably, subjective report could therefore be considered a more valid measure of disability and distress in FMD. Reliance on patient reported outcomes allows for less obtrusive remote assessments (via web-based forms and post), which in turn may limit the introduction of artificial aspects to outcome measurement (e.g. the desire to please the clinician) [37]. To support the battery of patient reported outcomes, we will report and analyse NHS Digital data on episodes of care. This non-patient reported and objective outcome will be considered a proxy measure of improvement.

If the trial intervention proves to be clinically and cost effective, there will be a strong argument in support of commissioning specialist services for people with FMD, and rolling out the intervention across the NHS. A positive result should also have an international impact, providing an impetus for the development of FMD services beyond the UK. Although this is a trial of neurology and physiotherapy treatment, the data gathered on the usefulness of the trial intervention may well have important implications for the clinical practice of occupational therapists, psychiatrists and psychologists working with people with functional neurological disorder.

## Trial status

Participant recruitment began on 15 November 2018 and is ongoing.

## Data Availability

Not applicable.

## Abbreviations

CGI-I: Clinical Global Impression Scale of Improvement
CSRI: Client Service Receipt Inventory
DMEC: Data Monitoring and Ethics Committee
FMD: Functional Motor Disorder
GLM: General Linear Model
HES: Hospital Episode Statistics
NHS: National Health Service
PHQ-15: Patient Health Questionnaire 15
Physio4FMD: A randomised controlled trial of physiotherapy for functional motor disorder
QALY: Quality Adjusted Life Year
RCT: Randomised Controlled Trial
SF36: Short Form 36
TSC: Trial Steering Committee
WPAI: Work Productivity and Activity Impairment scale
